# The benefits of in-group contact through physical activity involvement for health and well-being among Korean immigrants

**DOI:** 10.3402/qhw.v9.23517

**Published:** 2014-05-28

**Authors:** Junhyoung Kim, Jinmoo Heo, Jun Kim

**Affiliations:** 1 Department of Recreation, Parks, and Leisure Services Administration, Central Michigan University, USA; 2 Department of Recreation, Park and Tourism Sciences, Texas A&M University, College Station, Texas, USA

**Keywords:** Physical activity, health, well-being, culture

## Abstract

This qualitative study is designed to examine the benefits of physical activity involvement with members of the same ethnic group. For this study, Korean immigrants who were members of Korean physical activity clubs such as badminton and tennis were selected as participants. Using a constructive grounded theory methodology, three themes were identified as benefits of physical activity involvement: (1) the experience of psychological well-being, (2) the creation of a unique cultural world, and (3) the facilitation of physical activity involvement. The findings of this study suggest that Korean immigrant participants gained various social, cultural, and psychological benefits by engaging in activities with other Korean immigrants.

According to the perspective of social psychology, individuals tend to interact with others who have similar cultural and ethnic backgrounds and evaluate them in a more positive manner than those of a different culture and ethnicity (Brewer, 2007; Byrne, 1971). In a multicultural situation, individuals may easily acknowledge cultural and ethnic differences, and based on culture and ethnicity, they favor their in-group members (those who are similar to themselves) and denigrate out-group members (those who are dissimilar to themselves) (Pettigrew & Tropp, 2000). According to social identity theory, this in-group favoritism reflects people's desire to maintain a positive, in-group based interaction, which forms social identity (Pettigrew & Tropp, 2006). Social identity theory suggests that in an intercultural context, an ethnic group whose members share a similar cultural membership has a tendency to remain with, support, and favor those individuals with a similar culture and ethnicity. In particular, immigrants experience cultural exchanges and cultural negotiation during acculturation and may tend to maintain their cultural and ethnic identity. Safdar, Lay, and Struthers (2003) suggested that immigrants strive to maintain and develop in-group contact and interactions and express in-group favoritism. A possible reason for this phenomenon is that through in-group contact, immigrants are able to share their own cultural elements such as language, custom, and tradition, and express their experiences and feelings associated with acculturation.

One example of in-group contact among immigrants is engagement in personally meaningful activities with others who have the same ethnic and cultural backgrounds. Multiple researchers have demonstrated that members of an ethnic group pursue leisure activities that are associated with their own culture, and that they engage in these activities with other members of the same ethnic group (Iwasaki & Bartlett, 2006; Kim, Kleiber, & Kropf, 2001). The findings of these studies suggest that members of an ethnic group, including immigrants, form their own cultural boundaries through activities. In one study researchers found that Aboriginal individuals with diabetes engaged in culture-related activities and experienced cultural strength and positive feelings generated by in-group activity participation (Iwasaki, Bartlett, Gottlieb, & Hall, 2009).

Kim et al. (2001) observed that older Korean immigrants express an emotional and cultural attachment to their own culture and participate in activities with other Korean immigrants. By interacting with other Korean immigrants, older Korean immigrants maintained and developed their cultural and ethnic identities and experienced cultural strength. In a study of Chinese graduate students’ leisure behaviors, Li and Stodolska (2006) mentioned that the students tended to engage in activities with others who had the same ethnic and cultural characteristics. These studies suggest that immigrants tend to participate in activities with others of the same ethnic and cultural backgrounds.

Unfortunately, little information exists regarding the reasons why immigrants tend to engage in their activities with members of their own ethnic group, and what benefits they can gain through in-group activity. We used a qualitative study to determine the benefits of activity involvement in an in-group activity setting. For this study, we selected Korean immigrants who were members of Korean badminton and tennis clubs in order to examine a context of physical activity involvement. This was done for multiple reasons. First, Korean immigrants are one of the fastest growing ethnic groups in the United States (Lee & Woo, 2013). Some researchers have mentioned that there is little research on health-promoting behaviors related to activity participation among Korean immigrants (Berkman & Ko, 2010). Korean immigrants have a greater tendency to maintain their cultural values and beliefs than other ethnic groups (Lee & Yoon, 2011). Because of this tendency, they may express their desire to engage in their activities with other Korean immigrants.

## Literature review

### In-group vs. out-group and cultural identity among immigrants

To belong to an in-group, individuals share similar characteristics such as culture, race, and ethnicity, and psychologically identify themselves as being a member in a group (Stephan, Ybarra, & Bachman, 1999). The explanation for this phenomenon is that individuals feel more comfortable and less psychologically distressed when they interact with others of a similar ethnicity and race (McPherson, Smith-Lovin, & Cook, 2001). Otten and Moskowitz (2000), who studied 29 university students, found that students were more likely to express positive traits about an in-group member (similar to themself) than an out-group member (dissimilar to themself). The results of Otten and Maskowitz's study support evidence for the existence of a positive in-group stereotype and a tendency for in-group contact and interactions. This finding also indicates that when individuals interact with in-group members, they easily develop group cohesion and experience psychological comfort.

Previous studies have explored the effects of in-group contact on psychological well-being (Frable, Pratt, & Hoey, 1998; Sanchez & Garcia, 2009). The authors of these studies suggest that interaction with members of the same ethnic group is positively associated with psychological well-being among ethnic minorities (Frable, Pratt, & Hoey, 1998; Sanchez & Garcia, 2009). In a study of biracial individuals conducted by Sanchez and Garcia (2009), the presence of similar individuals in their daily life would predict greater daily well-being among them. This study indicates that the interaction among members of the same ethnic group functions as a facilitator of psychological well-being among ethnic minorities. Members of the same ethnic group share a similar cultural identity and cultural components such as language, custom, and social norms, which facilitate positive in-group contact and interactions (Postmes & Branscombe, 2002; Yip, 2005).

Immigrants’ cultural and ethnic identity may influence and be influenced by their acculturation process. Acculturation refers to a process of being accepted into and navigating new cultural components such as language, values, beliefs, customs, and social norms (Berry, 1980; Mena, Padilla, & Maldonado, 1987). A positive aspect of acculturation is that immigrants are able to learn new cultural perspectives, gain cultural knowledge, and develop a sense of cross-group friendship. In general, acculturation is often a stressful and distressing process for immigrants because they encounter various adaptation challenges such as differences between two cultures, cultural conflicts, language barriers, and interracial tensions (Berry & Kim, 1988; Park & Rubin, 2012). Such adaptation challenges are negatively associated with health and well-being among immigrants (Ra, Cho, & Hummer, 2013). To deal with adaptation challenges, immigrants may prefer to interact with members of the same ethnic group because they share the same cultural identity and immigration experiences that occurred during the acculturation process.

### Physical activity and Asian immigration

Substantial studies have demonstrated that acculturation is positively associated with physical activity involvement among Asian immigrants (Kandula & Lauderdale, 2005; Lee, Sobal, & Frongillo, 2000). In a systematic examination of 44 articles associated with acculturation and physical activity, Gerber, Barker, and Puhse (2012) found that more than 50% of all studies showed that acculturation is positively associated with physical activity involvement. Kandula and Lauderdale (2005) examined the relationship between physical activity and acculturation among Asian Americans. They found that Asian American immigrants are more likely to be physically inactive than individuals in a host community. These studies suggest that if immigrants are more acculturated to a new society, they are more likely to engage in various types of physical activities.

Stodolska and Yi-Kook (2005) suggested that immigrants perceived challenges to engagement in activities because of adaptation difficulties. These authors provided three main factors that negatively influenced participation in leisure activities: (1) lack of money and time, (2) deficient communication skills related to language barriers, and (3) limited social support. They also suggested that because of adaptation difficulties, immigrants perceived more challenges when utilizing physical activity resources and engaging in various leisure activities than other ethnic groups.

Some researchers provide evidence that linguistic barriers appear to be critical in preventing immigrants from participating in activities (Juniu, 2002; Yu and Berryman, 1996). In a study of immigrants by Rublee and Shaw (1991), the authors suggested that language difficulties negatively affect women immigrants’ participation in activities, which restricted them to mainly engage in home-oriented, passive, and child-care-related activities. Tcha and Lobo (2003) conducted a study of gender difference in physical activity participation related to acculturation among Korean immigrants in Australia. They found that female immigrants experienced higher levels of physical inactivity than males, which led to low acculturation. This study suggested that female immigrants might lack opportunities to interact with host individuals and perceive more adaptation challenges associated with acculturation. According to Afable-Munsuz, Ponce, Rodriguez, & Perez-Stable (2010), individuals in their study who were more acculturated to a new society increased participation in leisure-time physical activities and utilized more physical activity resources.

Previous literature suggests that interaction with the same ethnic group contributes to the psychological well-being of immigrants. Although many immigrants face challenges associated with acculturation, participation in physical activity appears to be critical in reducing adaptation challenges and improving health and well-being. It is expected that by engaging in physical activities with the same ethnic group, immigrants may gain various health-related benefits.

## Methods

We used a constructive grounded theory methodology to identify the benefits of physical activity involvement as a context of contact activity with the same ethnic group among Korean immigrants (Charmaz, 2004, 2006). Constructive grounded theory is beneficial for researchers because it provides multiple viewpoints of social contexts and social phenomena involved with participants’ values, beliefs, and feelings related to their personal life experiences (Charmaz, 2009). This also helps researchers to understand the cultural complexities of participants’ lives and experiences (Charmaz, 2006). In this study, by using constructive grounded theory, we were able to understand and analyse the meanings given to physical activity involvement through interactions with members of the same ethnic group among Korean immigrants.

### Participants

To respond to a qualitative research inquiry, we mainly focused on the positive experiences of participants who were members of ethnic group based activities such as the Korean tennis and badminton clubs. For this study, the criteria required were that individuals (1) had moved to the United States from South Korea, (2) were currently members of the Korean tennis and badminton clubs, and (3) were over 18 years old. The researchers collected information on where the Korean club activities were held and visited the activity sites. With permission from the president of each activity club, we had an opportunity to introduce our study to participants before the practice started. After a brief introduction of this study, potential participants expressed an interest in this study and in 2 weeks we contacted them individually. We set up a meeting in order to provide potential participants with research information such as the purpose of the study, confidentiality, and the participants’ rights to withdraw from the study at any time. The university institutional review board approved these procedures. Finally, 13 participants in the tennis and badminton clubs voluntarily engaged in this study. Ten participants were members of the badminton clubs and the average time of their participation in badminton was 12 years. Participants who were members of the tennis club had approximately 10 years of leisure experience. Out of 13 participants, 8 were men and 5 were women. The age range was from 34 to 65 and the average of their length of stay was 17 years.

In terms of theoretical saturation, according to Guest, Bunce, and Johnson (2006), 12 participants are generally an adequate number to reach saturation if they belong to a relatively homogenous group based on culture and ethnicity. This study used the constant comparative method suggested by Merriam (1998) and when we interviewed the 12th participant, we discovered that no new data had emerged. To fully reach a saturation point, we conducted one more interview and found that no additional information emerged.

### Data collection

The data were collected through in-depth interviews and, following consent, the interviews occurred at the place and time that was most convenient for each participant. Each interview lasted between 50 and 90 min. The research team developed semi-structured interview questions and applied the grand tour and mini tour interview strategy introduced by Spradley (1979). The interview started with grand tour questions such as: “Do you feel most comfortable speaking in English or Korean?”, “When did you become a member of the Korean activity club?” and “Tell me about your general experience in being a member of this club.” The interviews became more structured and focused on the benefits of physical activity involvement with the same ethnic group of members as mini tour questions. Examples were “Why did you participate in this activity?” “What benefits do you experience when you participate in this activity?” and “Did you have any challenges in participating in this activity because all of the participants are Korean?” At the end of the interview, the participants completed a brief demographic survey with questions regarding age, gender, length of stay, and marital status. We used pseudonyms for the participants throughout the research process.

### Data analysis

We conducted an analytic data analysis suggested by Glaser and Strauss (1967) under a structure of Charmaz's (2006) analytical approach. A three-step data analysis was performed which consisted of: (1) the initial phrase, (2) open coding involved with a line-by-line level of analysis, and (3) selective coding. In terms of the initial phrase, the second investigator created and produced raw data after each interview. With the creation of raw data, each investigator independently generated open coding and completed the process. The research team met regularly in order to share and discuss the open coding created by each investigator. During the meeting, we examined how the units of meaning from codes were compared with one another. A line-by-line level of analysis was followed for the second step. The research team categorized coding and generated implicit meanings of the emerging categories. With categorized coding, the research team identified causal relationships among categories and created sub-categories from the emerging categories. Selective coding was the final phase in which the investigators interpreted selected categories and produced the core themes and subthemes, including rich quotes and narrative descriptions. Data collection and data analysis were conducted concurrently, which enabled us to incorporate the core themes for subsequent in-depth interviews. During data analysis, when discrepancies among investigators emerged, we discussed them until they were resolved and where necessary, we shared identified themes with participants in order to clarify the discrepancies.

### Trustworthiness

We used various strategies to maintain quality and rigor of the data. The first strategy used was a member-checking process suggested by Lincoln and Guba (1985). Of the13 participants, nine voluntarily participated in the member-checking process. Based on the guidance suggested by Peterson, Papes, and Eaton (2007), we provided an opportunity for clarification and elaboration with a summary of the data analysis (e.g., themes and meanings) and they rated their satisfaction as “unsatisfactory” or “satisfactory.” Participants reviewed a summary of the themes and expressed that they were satisfied with our interpretations of the data. To improve credibility of the data, we used a back-translation process. Qualitative researchers have emphasized the value of back-translation when they conducted research related to culture and ethnicity to improve reliability of the data (Guillemin, Bomardier, & Beaton, 1993; Suh, Kagan, & Strumpf, 2009). We invited two graduate students fluent in English and Korean to participate in the back-translation process. With the guidance of Guillemin et al. (1993), we provided two graduate students with randomly selected paragraphs and allowed them to do the back-translation. In a few weeks, the second investigator had a meeting with them to validate the quality and conceptual equivalence of the translation. There were no requested changes. Last, each investigator was involved in data collection and data analysis and had expertise in conducting qualitative research.

## Results

Participants identified various benefits as a result of involvement in physical activities. In this article, we sought to capture the benefits of physical activity involvement because of in-group contact and interaction among members of the same ethnic group. Three main themes were identified as benefits: (1) the experience of psychological well-being, (2) the creation of a unique cultural world, and (3) a facilitation of physical activity involvement. These identified themes indicate that by engaging in physical activities with members of the same ethnic group, participants gained psychological, social, and cultural benefits, which contributed to their health and well-being.

### The experience of psychological well-being

The experience of psychological well-being was identified as the most salient theme that emerged from the data. All participants mentioned that they encountered adaptation challenges such as language barriers, discrimination experiences, feelings of isolation from the community, limited social networks, and cultural and ethnic differences. Because of such adaptation challenges, they said that they experienced psychological distress and loneliness. However, by participating in physical activities with other Korean immigrants, participants believed that their sense of psychological well-being increased through positive emotions and feelings, acculturation, and the ability to cope with acculturative stress.

All participants stated that they experienced positive emotions and feelings by interacting with other Koreans through physical activities. They believed that physical activity involvement provided an opportunity for them to share their personal life experiences that were associated with acculturation. Kim (male, 37) said that he created group cohesion and bonding with other participants because of their similar immigration experiences. As a result, he felt that playing badminton provided an opportunity to develop friendships, which helped deal with feelings of loneliness and depression. According to Lim (female, 51),

My husband lives in Korea and I am taking care of my children here for their education. I usually felt lonely and depressed because my English was not fluent and I did not have any friends here … after joining a badminton club, I made good friends who understood my situation as an immigrant and I really enjoyed playing badminton with them. I do not feel any loneliness or depression anymore because of my friends.

She also mentioned that after games, she joined social gatherings with other participants and had positive social interactions, which resulted in the expansion of her social networks.

Won (male, 65) said that he had depended on alcohol because he had encountered challenges to adapt to the host community. After interacting with other Korean immigrants through physical activity, he shared his life with other Korean immigrants and developed intimate and close relationships. As a result, he developed a strategy to deal with his negative feelings and reduced his level of acculturative stress associated with adaptation challenges.

Some participants who were members of other club activities with Americans made interesting comments on what differences existed between the two groups. They mentioned that they felt more psychological distress because of unfamiliarity with the culture in a multicultural setting. Kim (female, 34) mentioned that she was able to improve her English skills when she exercised with her American friends, but she preferred to play tennis and badminton with her Korean friends because she thought that she experienced an enhanced sense of happiness and positive mood with them. Lim (female, 51), who was a member of a local exercise center, said that it was difficult to make friends because of her limited English skills and cultural and ethnic differences. She also stated,

… I joined a yoga club and most of the participants were white women. They made friends easily and talked about lots of stuff. I just felt left out because I could not join any conversation because of my English, and it seemed that they did not initiate any conversations with me.

She felt uncomfortable with the fact that she could not develop a sense of belonging and connection in a group.

Sung (male, 52), who joined the Korean badminton club with his wife, said that positive feelings and emotions increased because his wife easily created friendships with other female Korean participants. He mentioned that his wife had a difficult time in adjusting to a new society and had experienced limited social relationships. After joining club activities together, they practiced together and had quality time with each other, which contributed to positive emotions and feelings.

In a context of physical activities, some participants gained valuable information related to immigration because they exchanged practical information and knowledge related to acculturation. They provided each other with a variety of information related to purchasing a home, visa-related issues, and house-related matters (e.g., plumbing, shopping, and moving). They thought that physical activity provided an opportunity for them to share immigration-related matters and gain a sense of cultural knowledge. According to Kim-J (male, 34),

After the game, we talked about a variety of immigration things and exchanged strategies to adapt to a new culture …. I gained knowledge and information related to immigration such as where to go when I was sick, or when I need some stuff. This was so helpful for me to ask some of the many questions I have about immigration.

He felt that without physical activity involvement he could not utilize resources and information related to acculturation, and that he gained a psychological comfort from the activity.

By engaging in activities with other Korean participants, some participants mentioned that they developed the ability to deal with adaptation challenges and reduced their level of acculturative stress. They used similar expressions such as “I reduced my stress related to acculturation,” “I became really happy when I came back home because after playing games, my stress was gone” and “Playing tennis with my Korean friends helps me reduce my stress.” It seems that they used activity involvement with other Korean immigrants as a resource for coping with acculturative stress.

Based on participants’ personal experiences, involvement in physical activity with members of the same ethnic group provides opportunities for Korean immigrant participants to have positive feelings and emotions, gain cultural knowledge, and reduce their level of stress associated with adaptation challenges. Our findings indicate that the participants were able to develop their personal relationships with other Korean participants, which may contribute to psychological well-being.

### The creation of a unique cultural world

The creation of a unique cultural world was another salient theme which emerged from the data. In a context of physical activity involvement, participants created their own unique world where they maintained their cultural identity and developed cultural and ethnic strength. They believed that they needed to practice their own cultural values and beliefs, and followed cultural norms when they interacted with other Korean club members. They thought that they preserved their cultural identity and because they were from a similar culture, they easily created a unique cultural and emotional bond with other Korean club members. Ha (male, 34) said that he lived in two different worlds: he followed Korean cultural values and beliefs when he played with Korean members, and he also sought to assimilate to new cultural perspectives.

According to all participants, language played an important role in creating their own world and maintaining their cultural identity. They spoke Korean, and even though a few participants felt comfortable speaking English, they continued to speak Korean and used their Korean names. Some participants commented that when they encountered people outside of the group, they tried to speak English because it was more appropriate to use English. Most participants said that they easily provided each other with constructive comments and feedback on activity skills and techniques, and exchanged a variety of information related to equipment and partner choices because they spoke the same language. They were able to elaborate on expressing their feelings and emotions when they played games. According to Ha (male, 34),

… when I made some mistakes while playing tennis with Americans, it made me feel so embarrassed and sorry. Also, with my limited English skills, it was difficult to explain my situation and exchange feedback …. Since I became a member of the Korean tennis club, I totally felt a sense of belonging and connectedness because we speak the same language, had a better understanding of each other, and supported each other. It was a totally different feeling, and I felt like I lived in two different worlds.

He also felt a sense of ethnic power and cultural strength because of the group dynamics generated by interacting with other Koreans.

Other cultural features that participants maintained were respect for older people, the use of honorific language, sharing food, and collectivistic behaviors. When they participated in activities, they said that they used honorific language when they talked to others who were older and bowed to each other to show respect. Sung (male, 52) stated that it was normal for him to practice Korean culture in the Korean tennis club and to use honorific language in order to show respect for older participants.

Participants also shared their food and beverages with others in and out of club activities. In particular, women participants prepared food and beverages for their fellow club members. Hong (female, 49) grew vegetables and fruits that were chemical free in her back yard, and brought them to share with other participants as part of their Korean culture. She said that she was happy to share a variety of vegetables and fruits with her club members. Ha (male, 34) said,

When my wife and son visited South Korea, many club members brought me a variety of Korean food that I was able to eat at home …. They regularly invited me to their places to have dinner together because they knew that I was a guy and could not cook very well.

Because Ha was temporarily living by himself, some of the club members might have been concerned that he would not be able to eat well. He also expressed an appreciation of Korean culture when club members shared a variety of things together.

In a show of collectivistic behavior, participants purchased the same equipment, clothing, and shoes to show group unity. They mentioned that there was no problem with sharing their equipment with others. Jung-w (female, 54) said that she always thought that this is “our team,” not “my team,” and she shared her equipment with others even though she purchased her own racket. With the establishment of friendship through club activities, the participants attended social gatherings during weekends to maintain and develop friendships. They developed intimate relationships and expanded their social networks through social gatherings, and it was observed that they applied their collectivistic ideas to their personal lives. Ha (male, 34) said that “after a game or during the weekend, we gathered at the park and Korean restaurants in order to socialize with each other, and I felt that they were like real family.”

These examples show that participation in club activities with members of the same ethnic group allowed participants to create their own unique world where they practiced and followed their cultural values and beliefs. In a context of club activities, they preserved their cultural identity and developed interpersonal relationships, which resulted in a unique cultural world. Our study indicates that such a creation of ethnic and cultural boundary helps participants to feel secure and improves their health.

### The facilitation of physical activity involvement

All of the participants had perceived some difficulties regarding engagement in activities because of adaptation challenges. The presidents of two Korean activity clubs were aware of the value of physical activity involvement for Korean immigrants and realized that acculturation often restricted them to engage in these activities. The primary purpose of these activities was to encourage Korean immigrants to pursue healthy leisure lifestyles and improve their health and well-being. According to Ha (male, 34), who was the president of the Korean badminton club,

I realized that many Korean immigrants were physically inactive and thought that I should think about how to encourage their involvement in physical activity. I was a professional badminton player in Korea and wanted to create a Korean badminton club …. Currently, there are over 30 immigrants who have become members and more Korean immigrants have expressed their interests in participating in this activity.

He mentioned that he received positive comments and feedback from participants, and that they became physically active and gained various physical, social, and psychological health benefits.

Participants mentioned that they had been physically inactive and experienced some difficulties when utilizing resources for physical activity involvement because of adaptation challenges. Before joining the club activities, participants said that they pursued sedentary activities such as watching Korean dramas and movies and reading books. However, after becoming members of the Korean club activities, they believed that they became physically active and gained health benefits such as physical and psychological fitness. They thought that participating in activities with other Korean immigrants served as a motivation to pursue an active lifestyle. Jung-W (female, 54) stated,

I was so happy to hear that there was the Korean badminton club because I wanted to play badminton. Even though I found an international badminton club at the local gym, there were participants from China, India, and Taiwan …. I was not encouraged to go over there to play badminton because of communication issues. That's why I was so happy to play the game with other Koreans, and it was a good motivation for me.

She also mentioned that she was motivated to master advanced skills and techniques related to badminton.

Some male participants said that they had experienced a limited scope of activity choices. They usually took a walk and went running at the park by themselves because it was a challenge to find activity partners and engage in physically demanding activities. Jung (male, 56) said that he used a trail to exercise and often played tennis at the gym with members of other ethnic groups. He mentioned that this did not fully satisfy his need for involvement in physical activity because he felt that there were some ethnic boundaries and adaptation challenges. After being a member of the club activities, he appreciated the opportunity to participate in physical activities where he developed a sense of belonging and connectedness.

A few female participants who tended to engage in less physically demanding activities mentioned that they had had a lack of interest in joining the club activities because engaging in physical activities was not among their leisure preferences. However, they became enthusiastic players because they were encouraged to participate in physical activities by other Korean immigrants and discovered that playing badminton and tennis brought enjoyment and fun to their lives. Gmar (female, 46) mentioned,

My close friend was encouraging me to join the activity and I had been reluctant to play any sports because I knew that I was not good at sports …. I did not realize that I had the ability to learn advanced skills and became a good player. I am fully determined to play badminton and it makes me happy.

She also felt that playing with other Korean participants was enjoyable and fun because she did not have many opportunities to interact with them.

Some participants developed intimate friendships and provided encouragement and emotional support for each other through activities. Based on their friendships, participants encouraged each other to participate in physical activities and provided constructive feedback on the improvement of their skills and techniques. Some female participants mentioned that they contacted each other on a daily basis and, aside from their regular practice time, they gathered individually to develop their own skills and techniques and encourage each other during the daytime. As a result, they created social, emotional, and psychological attachments to each other and to the activities.

Based on these statements, we observed that participants faced challenges associated with adaptation that restricted them from participating in various physical activities. With the creation of club activities, they were encouraged to engage in activities and they created a social, psychological, and emotional support system among participants.

## Discussion

We intended to explore what benefits Korean immigrants gain as a result of involvement in physical activity such as tennis and badminton with other Korean immigrants. In this study, as members of Korean club activities, Korean immigrant participants obtained health-related benefits such as psychological well-being, the creation of their own unique cultural world, and active engagement in physical activities. The findings of this study indicate that physical activities with members of the same ethnic group produce an opportunity for Korean immigrants to experience social, cultural, and psychological benefits. Such positive outcomes may contribute to health and well-being among Korean immigrant participants.

According to social psychologists, individuals tend to interact with and express favoritism toward others who are of a similar race or ethnicity (McPherson et al., 2001). Older Korean immigrants tend to interact with other Korean immigrants and express a strong sense of cultural and emotional attachment to their culture (Kim et al., 2001). This social and psychological aspect of in-group favoritism and the tendency of in-group interactions can explain why Korean immigrant participants organized their own sports clubs among other Korean immigrants. Regarding positive outcomes, previous studies have demonstrated that ethnic minorities gained health benefits as they interacted with members of their own group in their daily lives (Frable, Pratt, & Hoey, 1998; Sanchez & Garcia, 2009). Yip (2005) suggested that if a group of members possesses the same cultural identity (e.g., language, social norms, and custom), they are likely to positively interact with each other, which leads to psychological well-being. Thus, Korean immigrant participants increased they psychological well-being and health because they engaged in physical activities with other Korean immigrants.

Researchers have emphasized the value of intergroup contact through leisure activities for immigrants’ acculturation (Kim, 2012; Suh & Kim, 2011). These authors found that by interacting with other ethnic groups through leisure activities, immigrants established and developed a sense of friendship and gained cultural knowledge, which improved cultural and ethnic understandings. This study provides unique insights into the value of club activities that consist of members of the same ethnic group. The findings suggest that in a setting of intragroup activity, Korean immigrant participants gained social, cultural, and psychological benefits. This study suggests that it may be important for leisure professionals to not only promote intergroup contact and interactions through leisure, but to organize and provide activity programs that allow immigrants to foster intragroup interactions and contact.

The acculturation process is often stressful and psychologically distressing to immigrants as they encounter various adaptation challenges such as major lifestyle changes, cultural differences, differences in social norms, discrimination experiences, and language barriers (Mio, Barker-Hackett, & Tumambing, 2006; Stodolska, 1998). Berry, Poortinga, Segall, and Dasen (2002) suggested that the acculturation process is negatively associated with health and well-being among immigrants. This study shows that Korean immigrant participants developed the ability to deal with acculturative stress and improve psychological well-being by engaging in activities with other Korean immigrants. This also suggests that involvement in physical activity with the same ethnic group serves as an important vehicle for improving health and well-being among immigrants.

Researchers provide evidence that leisure-time physical activity plays an important role in improving positive feelings and emotions and reducing negative psychological symptoms (Blumenthal, Williams, Needels, & Wallace, 1982; Iso-Ahola & Park, 1996; Stathi, Fox, & McKenna, 2002). The findings of this study expand the idea that leisure-time physical activity that occurs among Korean immigrant participants helps them develop positive feelings and emotions, reduce a sense of loneliness and acculturative stress, and develop the ability to deal with adaptation challenges. This research also suggests that physical activities provided a positive environment in which Korean immigrant participants perceived a psychological comfort zone, which contributed to positive feelings and emotions among participants.

In a recent study of older Korean immigrants conducted by Kim and Kim (2013), older Korean immigrants engaged in a volunteering activity by helping other Korean immigrants adapt to a host community, which facilitated acculturation. In this study, in a context of physical activity, Korean immigrant participants exchanged practical information and knowledge related to acculturation and provided support systems for each other, which may lead to acculturation. This information also indicates that Korean immigrant participants may gain cultural knowledge and promote cultural and ethnic understandings by exchanging information about of adaptation.

The authors of previous studies have demonstrated that immigrants experienced lower involvement in physical activity than the host individuals because of adaptation challenges (Kandula & Lauderdale, 2005; Lee et al., 2000). One critical factor that prevented immigrants from participating in physical activities was that immigrants experience linguistic barriers (Juniu, 2002). In a context of physical activity with the same ethnic group, Korean immigrant participants may reduce language barriers, easily create cultural and emotional bonding, and develop friendships. Korean immigrant participants who underutilized physical activity resources were encouraged to engage in activities by other Korean immigrants, which increased their motivation for involvement in physical activity.

The participants in this study demonstrated that a unique social world was developed as a result of participating in the club activity. Stebbins’ (1992) serious leisure theory may help understand the behavior of Korean immigrants in this study. A tendency to form a unique cultural world or subculture around the activity is one of the characteristics of serious leisure. When individuals are engaged in serious leisure, a collective commitment is evident around the pursuit, and this commitment often leads the group to reach a common goal. The participants in our study expressed a collective form of commitment, and their language and cultural background facilitated links among the participants. Sharing food with club members, speaking Korean during the activities, and using honorific language were common practices among the participants that provided a framework for creating a unique cultural world.

## Limitations and need for future research

There are several limitations that we should address. First, we focused on the benefits of physical activity involvement with the same ethnic group among Korean immigrants. By interacting with members of their own ethnic group, participants may encounter some challenges such as a lack of opportunities to interact with members of other ethnic groups and low acculturation. It may be beneficial for future research to explore the dynamics of intergroup contact vs. intragroup contact activity among participants. Second, each participant may have a different level of acculturation and demographic information (e.g., education, gender, and socio-economic status). If future researchers examine the relationship among physical activity involvement, level of acculturation, and demographic information, they may provide insightful information and knowledge for this field. Also, this study was designed to capture the benefits of physical activity involvement because of in-group contact and interaction among Korean immigrants. Future research is needed to create an outreach program related to physical activities that help other immigrants adapt to a new society and culture. Finally, this study limited the scope of participants to Korean immigrants who were members of Korean club activities. Members of other ethnic groups may organize and engage in various activities among their own members. It is necessary to examine how members of other ethnic groups gain benefits as a result of activity involvement.
